# Obatoclax inhibits SARS-CoV-2 entry by altered endosomal acidification and impaired cathepsin and furin activity in vitro

**DOI:** 10.1080/22221751.2022.2026739

**Published:** 2022-02-10

**Authors:** Binli Mao, Vu Thuy Khanh Le-Trilling, Kai Wang, Denise Mennerich, Jie Hu, Zhenyu Zhao, Jiaxin Zheng, Yingying Deng, Benjamin Katschinski, Shilei Xu, Guiji Zhang, Xuefei Cai, Yuan Hu, Jianwei Wang, Mengji Lu, Ailong Huang, Ni Tang, Mirko Trilling, Yong Lin

**Affiliations:** a Key Laboratory of Molecular Biology of Infectious Diseases (Chinese Ministry of Education), Department of Infectious Diseases, The Second Affiliated Hospital, Institute for Viral Hepatitis, Chongqing Medical University, Chongqing, People’s Republic of China; b Institute for Virology, University Hospital Essen, University of Duisburg-Essen, Essen, Germany; c Department of General Surgery, The Third Affiliated Hospital, Sun Yat-Sen University, Guangzhou, People’s Republic of China; d Department of Immunology, College of Basic Medicine, Chongqing Medical University, Chongqing, People’s Republic of China

**Keywords:** Obatoclax, SARS-CoV-2, endocytosis, membrane fusion, MCL-1

## Abstract

*Coronavirus disease 2019* (COVID-19) caused by the emerging *severe acute respiratory syndrome coronavirus 2* (SARS-CoV-2) has set off a global pandemic. There is an urgent unmet need for safe, affordable, and effective therapeutics against COVID-19. In this regard, drug repurposing is considered as a promising approach. We assessed the compounds that affect the endosomal acidic environment by applying human angiotensin-converting enzyme 2 (hACE2)- expressing cells infected with a SARS-CoV-2 spike (S) protein-pseudotyped HIV reporter virus and identified that obatoclax resulted in the strongest inhibition of S protein-mediated virus entry. The potent antiviral activity of obatoclax at nanomolar concentrations was confirmed in different human lung and intestinal cells infected with the SARS-CoV-2 pseudotype system as well as clinical virus isolates. Furthermore, we uncovered that obatoclax executes a double-strike against SARS-CoV-2. It prevented SARS-CoV-2 entry by blocking endocytosis of virions through diminished endosomal acidification and the corresponding inhibition of the enzymatic activity of the endosomal cysteine protease cathepsin L. Additionally, obatoclax impaired the SARS-CoV-2 S-mediated membrane fusion by targeting the MCL-1 protein and reducing furin protease activity. In accordance with these overarching mechanisms, obatoclax blocked the virus entry mediated by different S proteins derived from several SARS-CoV-2 variants of concern such as, Alpha (B.1.1.7), Beta (B.1.351), and Delta (B.1.617.2). Taken together, our results identified obatoclax as a novel effective antiviral compound that keeps SARS-CoV-2 at bay by blocking both endocytosis and membrane fusion. Our data suggested that obatoclax should be further explored as a clinical drug for the treatment of COVID-19.

## Introduction

The world is currently in the midst of a global pandemic of *coronavirus disease 2019* (COVID-19) caused by the emerging *severe acute respiratory syndrome coronavirus 2* (SARS-CoV-2) [1]. Several highly effective vaccines against SARS-CoV-2 infection have been approved and are currently applied in various countries [2]. However, due to limited vaccine supplies and unsuccessful vaccine programs in rural, developing, and politically unstable regions, SARS-CoV-2 is still raging in various regions. Therefore, it is crucial to develop effective antiviral agents against SARS-CoV-2 infections to mitigate the consequences of the COVID-19 pandemic.

SARS-CoV-2 belongs to the *Sarbecovirus* subgenus of the *Coronaviridae* family. SARS-CoV-2 has a 30 kb unsegmented, single-stranded positive-sense RNA genome, which, in addition to an array of non-structural proteins, encodes the four principal structural proteins, spike glycoprotein (S), nucleocapsid protein (N), matrix glycoprotein (M), and envelope protein (E) [3,4,5]. Through its receptor binding domain (RBD), the S protein recognizes its receptor the human angiotensin-converting enzyme 2 (hACE2) [3], thereby initiating viral entry fusing the virus envelope with host cell membranes.

Many enveloped viruses are usually endocytosed and do not directly fuse with the cell surface [6]. Within endosomes, they depend on a tightly regulated acidic environment to activate proteases, which cleave and thereby maturate viral proteins such as the S protein, triggering the fusion of the viral envelope with the endosomal membrane. This fusion releases the viral genome within the nucleocapsid into the cytoplasm to initiate the replication cycle [7,8]. The high structural and genetic similarities between SARS-CoV-2, SARS-CoV, and MERS-CoV [9,10] suggested that the endosomal pathway might represent a druggable Achilles’ heel applicable for repurposing drugs against SARS-COV-2 infections.

Membrane fusion is indispensable for SARS-CoV-2 entry into host cells. For the fusion of the S protein, cellular proteases such as furin and trypsin-like serine proteases such as transmembrane protease serine 2 (TMPRSS2) are required [11,12]. These proteases cleave SARS-CoV-2 S at different sites: furin acts at the S1/S2 junction, while TMPRSS2 cleaves at the S2 site [13].

Based on our previous work [14,15] and aforementioned considerations, we investigated small compounds, such as niclosamide, glucosamine, bafilomycin A1, ouabain, chloroquine, and obatoclax, that affect endosomal acidification [16–21], and evaluated their protective ability against SARS-CoV-2 infections *in vitro*. Moreover, we further elucidated the underlying molecular mechanisms of obatoclax against the viral infection.

## Materials and methods

### Cell culture

Calu-3, A549, Caco-2, HEK293T, and HEK293T-hACE2 cells were maintained in our laboratories. All the cells were cultured in Dulbecco’s Modified Eagle’s Medium (DMEM; Gibco, Rockville, USA) supplemented with 10% fetal bovine serum, 100 U/mL penicillin–streptomycin, and 1× Minimum Essential Medium (MEM) Non-Essential Amino Acids (NEAA) solution at 37 °C in 5% CO_2_.

### Plasmids, inhibitors, and other reagents

Plasmids expressing GFP-S and hACE2 have been previously described [22]. The plasmid expressing TMPRSS2 was kindly provided by Prof. Yan Huan (Wuhan University, China). The plasmid with MCL-1 expression was constructed on empty vector pCMV-10 by General Biol Company (Chuzhou, China). Chloroquine (C6628) and N-acetyl-D-glucosamine (GlcN; A3286) were purchased from Sigma-Aldrich (Burlington, MA, USA) and both dissolved in sterile water. Aloxistatin (E64d; HY-100229), bafilomycin A1 (HY-100558), CA074-Me (HY-100350), chlorpromazine (HY-B0407A), MDL 28170 (HY-18236), niclosamide (HY-B0497), ouabain (HY-B0542), and TW-37 (HY-12020) were obtained from MedChemExpress (New Jersey, NJ, USA) and all dissolved in dimethylsulfoxide (DMSO). Obatoclax (GX15-070) was purchased from Selleckchem (Houston, TX, USA) and dissolved in DMSO. LysoTracker Red (Thermo Fisher, L12492) was used as previously described [7].

### SARS-CoV-2-S-pseudovirus system

SARS-CoV-2 pseudoviruses based on the HIV-1 backbone and including the complete S gene from the SARS-CoV-2 strain Wuhan-Hu-1 (GenBank: MN908947) were generated (S-pseudovirus) as previously described [22]. Briefly, HEK293 T cells were co-transfected with recombinant SARS-CoV-2-S plasmid green fluorescent protein (GFP)-S and pNL4-3.Luc.R-E – using Lipofectamine 3000 transfection reagent (Invitrogen, Rockville, MD, USA). The medium containing SARS-CoV-2 pseudoviruses was collected at 48–72 h of transfection and filtered through a 0.45 mm filter. The titres of pseudoviruses were calculated by determining the number of viral RNA genomes per mL of viral stock solution using RT-qPCR with primers targeted the LTR17. A known quantity of pNL4-3.Luc.R-E- vector was used to calculate standard curves. As to SARS-CoV-2-S pseudoviruses infection, a total of approximately 1.5 × 10^4^ Calu-3-hACE2, A549-hACE2, or Caco-2-hACE2 cells per well were seeded into 96-well plates and pre-treated with 50 μL of related drugs. After incubation with drugs for 2 h, the cells were infected with the pseudotyped viruses (3.8 × 10^4^ copies in 50 μL) for 72 h.

### The infections with clinical SARS-CoV-2 isolates

All authentic SARS-CoV-2 infection experiments were carried out in the BSL-3 laboratory of the University Hospital Essen, Germany. The SARS-CoV-2 wild-type (WT) strain B.1 and the variant Delta (B.1.617.2) were isolated from patient samples obtained in May 2020 and May 2021, respectively. For the isolation, permissive cells were incubated with virus-containing clinical nasopharyngeal swab samples until cytopathic effect (CPE) was observed. B.1 was amplified in Vero E6 cells, B.1.617.2 in Calu-3 cells. Viral titres were determined by 50% tissue culture infectious dose (TCID50) titration. The virus isolation has been approved by the ethics committee of the medical faculty of the University of Duisburg-Essen (20-9511-BO and 20-9512-BO). A total of approximately 3 × 10^4^ cells per well Calu-3 or Caco-2 cells were pre-treated with obatoclax (0.33, 1 μM, or 3.3 μM) for 1.5 h, followed by infection with SARS-CoV-2 (Multiplicity of Infection (MOI) = 0.1). At 2 h post-infection, the supernatant was removed and replaced by a fresh medium containing the respective amount of drug. At 24 h post viral infection, the supernatant of the infected cells was frozen for determination of progeny virus and the viral N and S protein levels in the infected cells were quantified by in-cell-ELISA (icELISA) [23].

### Luciferase reporter assay

A total of approximately 1.5 × 10^4^ Calu-3-, A549-, or Caco-2-hACE2 cells per well were seeded into 96-well plates for 8 h and then pre-incubated with related drugs for 2 h, followed by being infected with SARS-CoV-2-S pseudotyped viruses (3.8 × 10^4^ copies in 50 μL) for 24 h. After 72 h incubation post SARS-CoV-2-S-pseudovirus infection, cells were collected and lysed using 1 × lysis buffer (Promega, Madison, WI, USA). Subsequently, their luciferase activity was quantified using the Luciferase Reporter Assay System (Promega, E1500) according to the manufacturer’s protocol. Relative luminescence units (RLU) were measured using a GloMax microplate luminometer (Promega, Madison, WI, USA).

### Statistical analyses

Statistical analyses were conducted using GraphPad Prism 8 software (GraphPad Software Inc., La Jolla, CA, USA). Analysis of variance (ANOVA) with two-tailed Student’s t-test or nonparametric one-way ANOVA with a Dunn post-test was used to determine significant differences, and values of *p* < 0.05, were considered significant. All experiments were repeated independently at least 3 times.

## Results

### Obatoclax and other compounds that affect the acidic endosomal environment impair the entry of SARS-CoV-2

To investigate the effect of candidate compounds targeting endosomal acidification on SARS-CoV-2 infection, small chemical inhibitors such as niclosamide, glucosamine, bafilomycin A1, ouabain, and obatoclax were evaluated in human ACE2 transgenic HEK293 T cells (“HEK293T-hACE2”) infected with a recently described SARS-CoV-2 S protein-pseudotyped HIV-based reporter virus (“S-pseudovirus”) [22]. Chloroquine, a classical lysosomotropic agent that modifies endosome-lysosomal acidification, served as control. Cells were pre-treated with graded concentrations of the aforementioned compounds and then infected with the S-pseudovirus. At 72 h post-infection, the cytotoxicity of compounds and their capacity to prevent the infection were quantified. Niclosamide and glucosamine did not exhibit significant effects on the S-mediated virus entry in HEK293T-hACE2 cells (Fig. S1A-B), while ouabain did not show a favourable therapeutic index (Fig. S1C). Intriguingly, bafilomycin A1, chloroquine, and obatoclax showed significant inhibitory activity against the infection at nanomolar concentrations (Fig. S1D–F). A commercially available calorimetric “cell-counting” methodology indicated that bafilomycin A1 and ouabain exhibited greater cytotoxicity and/or anti-proliferative activity than obatoclax. Based on the favourable therapeutic index, obatoclax was selected for further molecular analyses.

**Figure 1.**
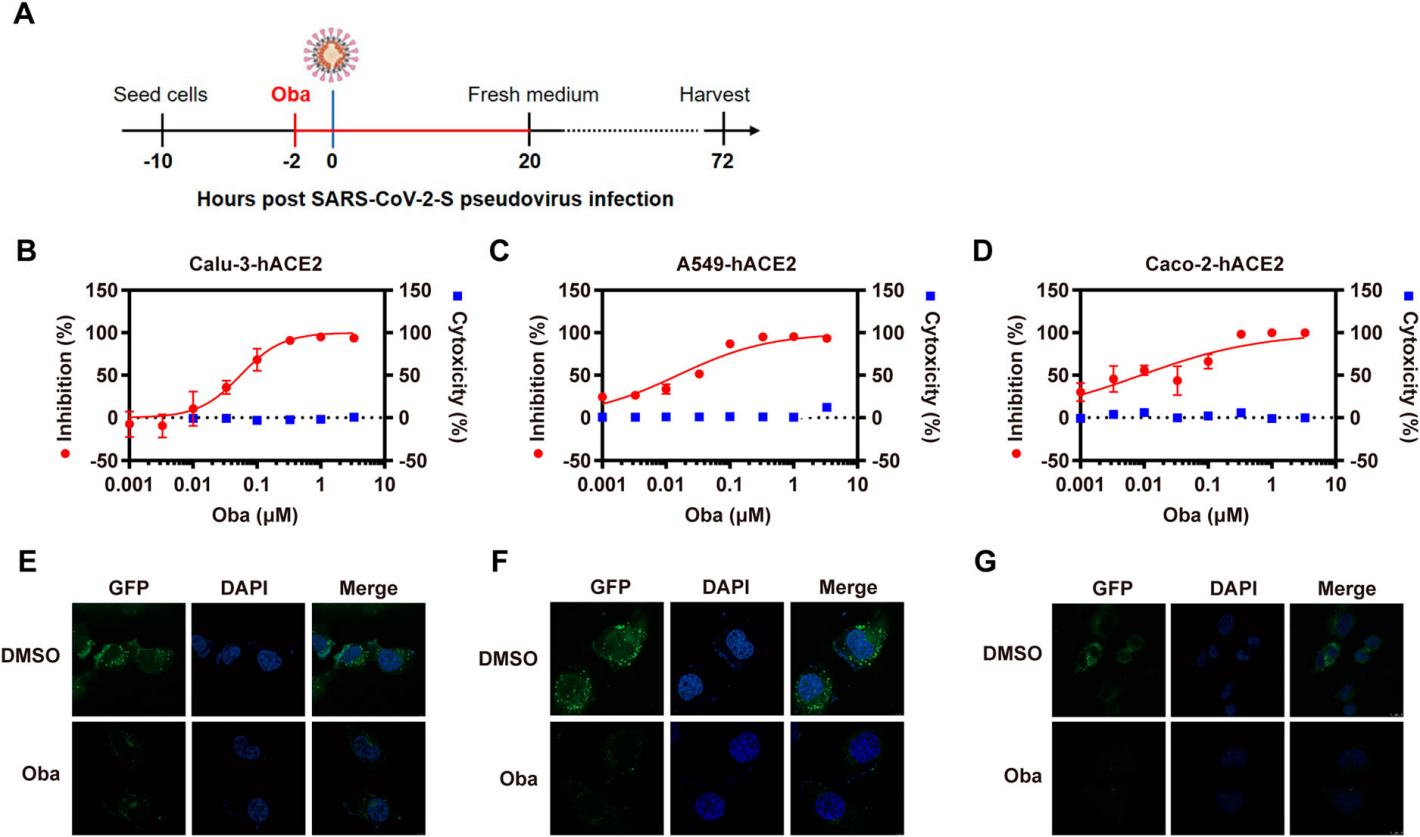
Obatoclax inhibits SARS-CoV-2 pseudoviral infections. (A) Schematic representation of the procedure of SARS-CoV-2 pseudoviral infection and obatoclax (Oba) treatment. (B–D) Human lung carcinoma cells Calu-3 or A549, as well as human colonic adenocarcinoma cells Caco-2, were pre-transfected with plasmid hACE2. At 72 h post transfection, Calu-3-hACE2, A549-hACE2, or Caco-2-hACE2 cells were pre-incubated with different concentrations of Oba (from 0.001 to 3.3 μM) or control DMSO for 2 h, followed by infection with SARS-CoV-2-S-pseudotyped viruses (7.6 × 10^5^ copies/mL) for 24 h. At 72 h post-pseudovirus inoculation, relative light units (RLUs) were detected using luciferase reporter assay and cell viability was measured using CCK-8 assay. These data were normalized to that of control DMSO. (E–G) Calu-3-hACE2, A549-hACE2, and Caco-2-hACE2 cells were pre-incubated with 0.2 μM Oba for 2 h and then infected with SARS-CoV-2-S-pseudotyped virusesas as (B–C). The fluorescent signal of viral spike expression was detected by confocal microscopy at 48 h post-virus infection. Scale bar: 10 μm; magnification: 630×. All these experiments were repeated at least three times.

Considering the restricted antiviral effects of chloroquine in human cells and to assess potential cell specificities, obatoclax was tested using hACE2 transgenic human Calu-3 (“Calu-3-hACE2”), A549 (“A549-hACE2”), and Caco-2 (“Caco-2-hACE2”) cells infected with the S-pseudovirus (Figure 1(A)). Compared to the control dimethyl sulfoxide (DMSO) treatment (Fig. S2A–C), we found that 330 nM obatoclax blocked >90% of viral entry in all three cell lines (Figure 1(B–D)). In contrast to its antiviral activity at low nanomolar concentrations, obatoclax concentrations below 3.3 µM did not exhibit cytotoxic effects. These findings were further corroborated and visualized by confocal microscopy, which indicated that the SARS-CoV-2 S protein expression is substantially decreased in cells upon obatoclax treatment compared to control-treated cells (Figure 1(E–G)). Additionally, we assessed the effect of obatoclax on the pro-inflammatory cytokine response, which is closely related to the severity of COVID-19 [24], in cells infected with SARS-CoV-2-S-pseudovirus. RT-qPCR assay results showed that the mRNA levels of five representative pro-inflammatory cytokines or chemokines including interleukin (IL)-6, IL-10, C-X-C motif chemokine ligand 10 (CXCL10/IP-10), C–C motif chemokine ligand (CCL) 2 (CCL2/MCP-1), and CCL3/MCP-1 MIP1α were not significantly different in obatoclax-treated A549-hACE2 cells, and that of IL-10, IP-10, and MCP-1 in Calu-3 cells (Fig. S3A-B). However, obatoclax significantly decreased the levels of IL-6 and MIP1α mRNA expression in the Calu-3-hACE2 cells, indicating that obatoclax has a certain anti-inflammatory effect on SARS-CoV-2 infection.Taken together, these data demonstrated that obatoclax potently inhibits the SARS-CoV-2 S-mediated entry into human cells.

### Potent antiviral effect of obatoclax in infections with clinical SARS-CoV-2 isolates

**Figure 2.**
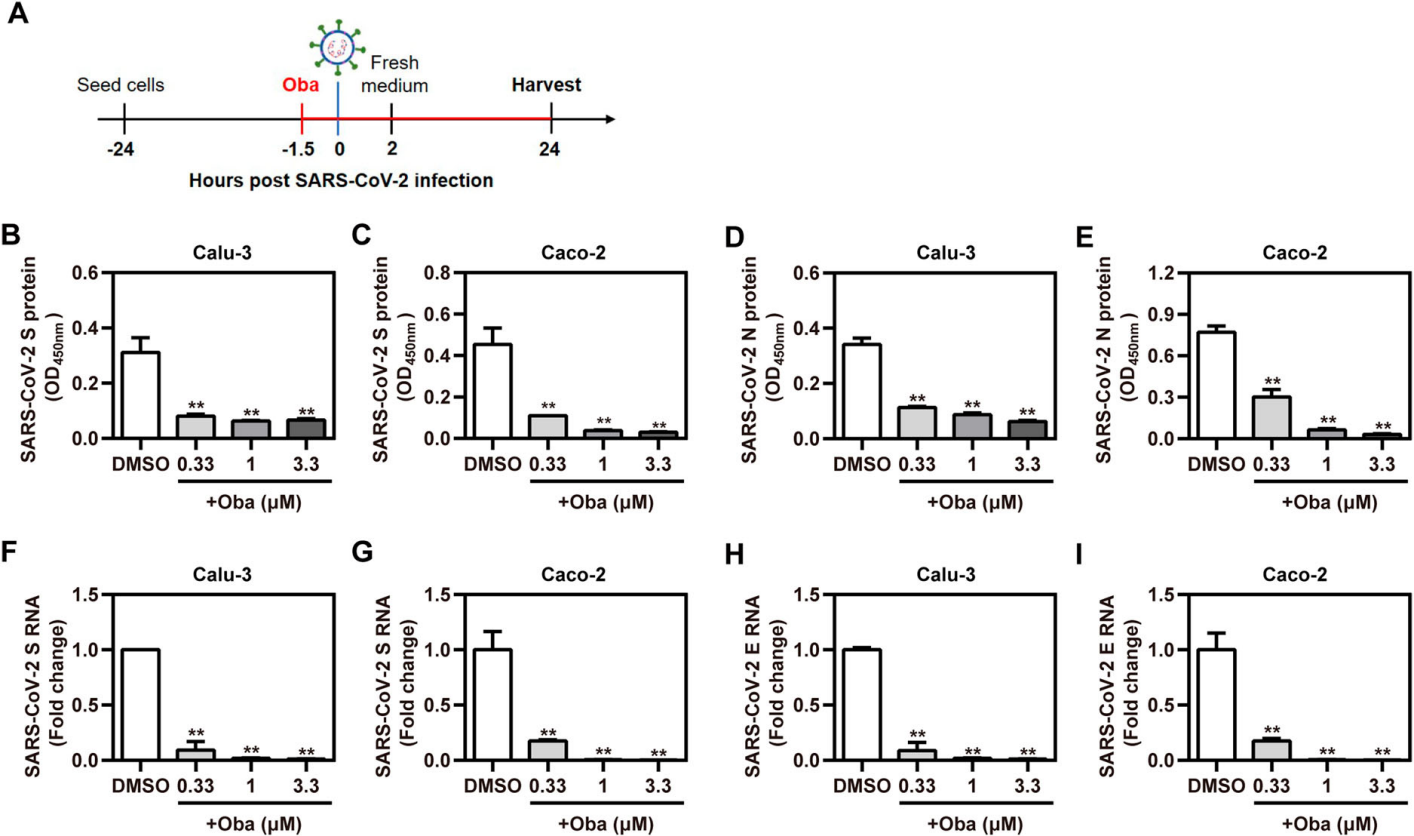
Obatoclax inhibits the infections with clinical SARS-CoV-2 isolates. (A) Schematic representation of the procedure of SARS-CoV-2 infection and obatoclax (Oba) treatment. (B-I) Calu-3 and Caco-2 cells were pre-treated with Oba at 0.33 μM, 1 μM, and 3.3 μM for 1.5 h and then infected with a clinical SARS-CoV-2 isolate wild-type strain B.1 (MOI  = 0.1). At 2 h post-viral infection, the supernatant was removed and replaced by fresh culture medium containing the respective amount of Oba. At 24 h post-virus inoculation, supernatant aliquots were frozen and viral S and N proteins in the infected cells were quantified by in-cell-ELISA (B-E). Viral progeny of SARS-CoV-2 was quantified from culture supernatants by realtime RT-qPCR targeting S and E gene (F-I). All these experiments were repeated at least three times. *P < 0.05; **P < 0.01; ns, not significant.

To evaluate the real antiviral activity of obatoclax against the infections with clinical SARS-CoV-2 isolates (the strain B.1), the abundance of the viral antigens in Calu-3 and Caco-2 cells, as well as the viral genome copies present in cell supernatants, were evaluated upon obatoclax treatment (Figure 2(A)). A recently described icELISA [23] was applied to quantify the abundance of the viral S and N protein in infected cells treated with the indicated graded obatoclax concentrations (0.33–3.3 μM). Obatoclax significantly and in a dose-dependent manner diminished the SARS-CoV-2-encoded S (Figure 2(B,C)) and N antigen (Figure 2(D,E)). The antiviral activity was further corroborated by determining the amounts of supernatant viral particles which was assessed by a diagnostic, dual-target RT-qPCR recognizing the S (Figure 2(F,G)) and E (Figure 2(H,I)) genes. Obatoclax significantly decreased viral replication in terms of the supernatant progeny virus. Thus, obatoclax shows a potent antiviral effect against authentic SARS-CoV-2 infections in human cells.

### Obatoclax inhibits the endocytosis of SARS-CoV-2

**Figure 3.**
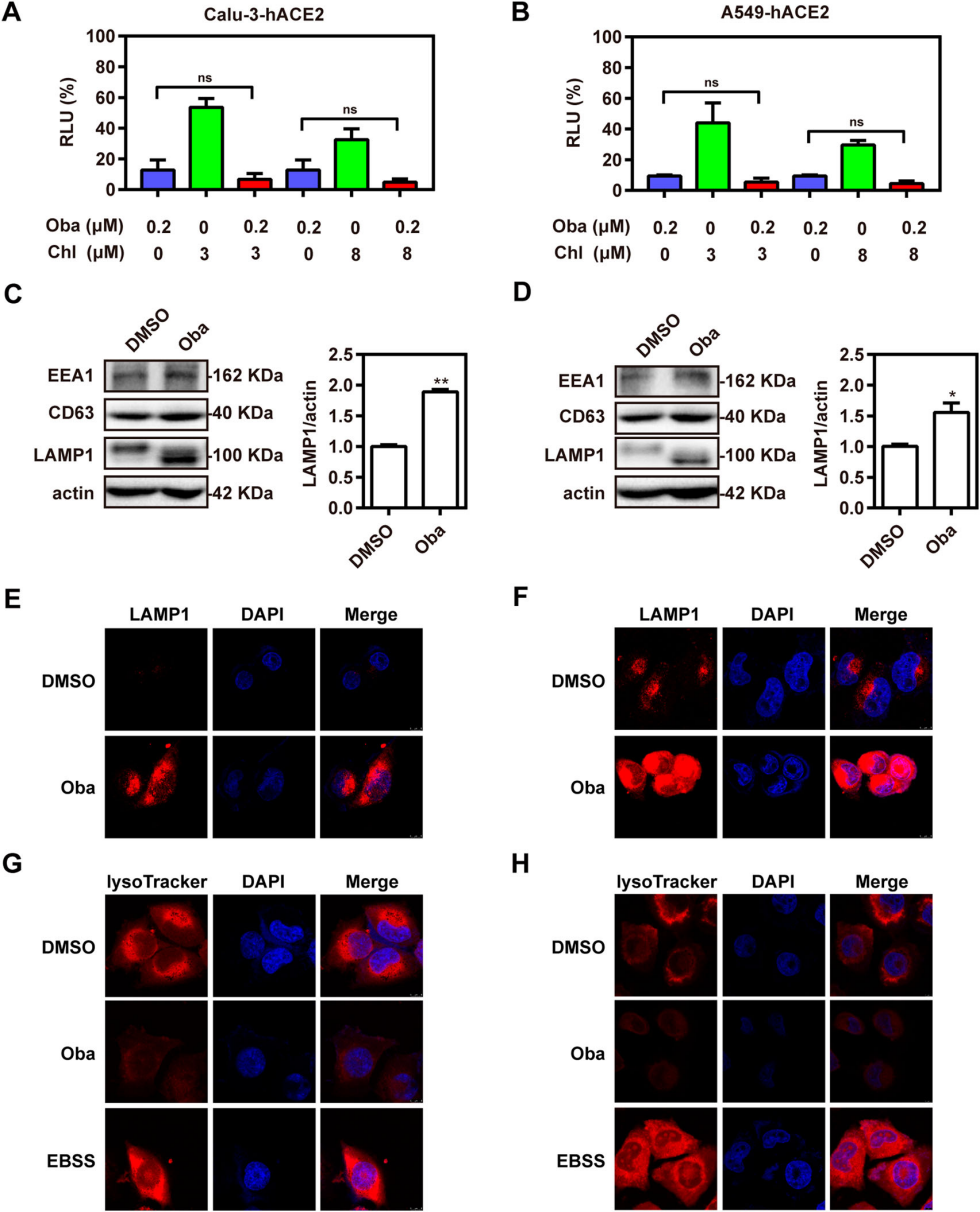
Obatoclax inhibits the endocytosis of SARS-CoV-2. Calu-3 (A) and A549 (B) cells were transfected with plasmid hACE2. At 72 h post transfection, Calu-3-hACE2 and A549-hACE2 cells were treated with 0.2 μM obatoclax (Oba) and 3 μM or 8 μM chlorpromazine for 2 h, followed by SARS-CoV-2-S-pseudotyped virus infection (7.6 × 10^5^ copies/mL) for 24 h. At 72 h post-pseudovirus inoculation, relative light units (RLUs) were detected using luciferase reporter assay and normalized to that of control DMSO. Calu-3-hACE2 (C) or A549-hACE2 (D) cells were treated with 0.2 μM Oba for 48 h. The levels of EEA1, CD63, and LAMP1 expression from cell lysates were detected by western blotting, using beta-actin as a loading control. The LAMP1 expression in Calu-3-hACE2 (E) and A549-hACE2 (F) cells were imaged by confocal microscopy. Calu-3-hACE2 (G) and A549-hACE2 (H) cells were treated with 0.2 μM Oba for 24 h, and stained using LysoTracker Red. Cells treated with Earle’s balanced salt solution (EBSS) and starved for 2 h were used as a positive control. The fluorescence intensity of LysoTracker Red was detected by confocal microscopy. Scale bar: 10 μm; magnification: 630×. All these experiments were repeated at least three times. * *P* < 0.05; ** *P* < 0.01; ns, not significant.

Given that obatoclax showed such potent antiviral activity in both the S-pseudovirus assay and authentic SARS-CoV-2 infections, we probed its molecular mechanism(s). Firstly, we assessed the effect of obatoclax on ACE2 expression *in vitro* using western blotting. Our results showed that obatoclax treatment did not cause an obvious change of ACE2 expression in Calu-3-hACE2 and A549-hACE2 cells (Fig. S4). Obatoclax, an organic base, can increase the pH of the acidic environment by entering the lysosomes, becoming protonated and trapped in them [17]. Obatoclax may inhibit the endocytosis of SARS-CoV-2 by impairing the lysosomal function. Chlorpromazine, a Food and Drug Administration (FDA)-approved inhibitor for clathrin-dependent endocytosis, shows inhibitory effects against SARS-CoV [25,26]. Thus, Calu-3-hACE2 and A549-hACE2 cells were treated with 0.2 μM obatoclax and 3 or 8 μM chlorpromazine to evaluate if both drugs act additively, which would point toward independent molecular mechanisms. However, no significant additive effect was observed (Figure 3(A,B) and data not shown). This negative finding prompted us to investigate if obatoclax, similar to chlorpromazine, may affect the endosomal pathway.

To this end, we evaluated the potential effects of obatoclax on the endosomal/lysosomal pathway by immunoblotting of known marker proteins. We found significantly increased abundances of the late lysosome marker *lysosomal-associated membrane protein 1* (LAMP1) in obatoclax-treated cells, while the expression of the early and late endosomal markers (*early endosome antigen 1* [EEA1] and CD63) remained unaltered (Figure 3(C,D)). Accordingly, confocal microscopy analysis supported the aforementioned immunoblot assay results confirming increased LAMP1 levels in obatoclax-treated cells (Figure 3(E,F)). Previous studies suggested that obatoclax could neutralize the acidic environment of endocytic vesicles and that this effect is associated with its antiviral activity against alphaviruses such as the chikungunya virus [17]. We hypothesized that obatoclax might increase LAMP1 expression by changing the endosomal acidification. To test this hypothesis, Calu-3-hACE2 and A549-hACE2 cells were treated with obatoclax and then incubated with LysoTracker Red, which specifically stains acidic membrane-surrounded compartments. The fluorescence intensity of LysoTracker Red staining was significantly decreased in obatoclax-treated cells (Figure 3(G,H)), suggesting that obatoclax inhibits endocytosis of SARS-CoV-2 entry by disturbing endosomal acidification.

### Obatoclax inhibits the activity of endosomal cysteine protease cathepsin L

**Figure 4.**
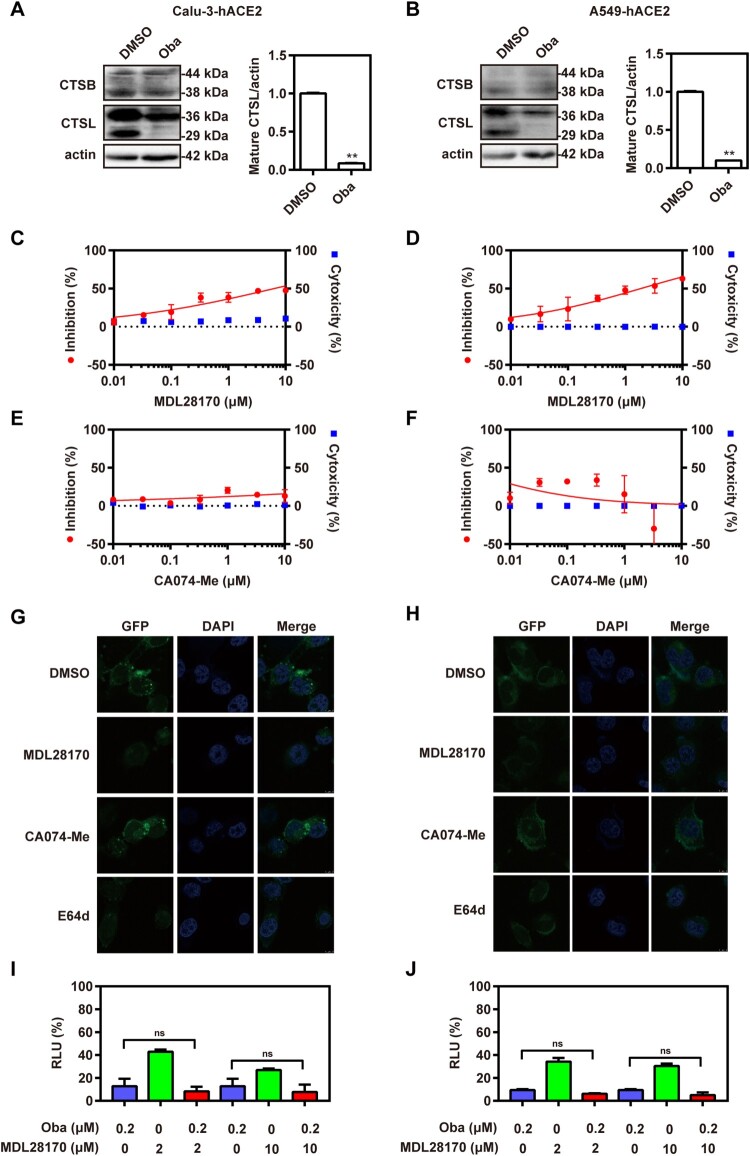
Obatoclax inhibits the activity of endosomal cysteine protease CTSL. Calu-3 and A549 cells were transfected with plasmid hACE2. At 72 h post transfection, Calu-3-hACE2 (A) and A549-hACE2 (B) cells were treated with 0.2 μM obatoclax (Oba) for 48 h. The levels of CTSB and CTSL expression from cell lysates were measured by western blotting, using beta-actin as a loading control. The ratios of mature CTSL: beta-actin were calculated by densitometry analysis. (C–F) Calu-3-hACE2 or A549-hACE2 cells were pre-treated with various concentrations of MDL28170 or CA074-Me from 0.01 μM to 10 μM or control DMSO for 2 h, followed by infection with SARS-CoV-2-S-pseudotyped viruses (7.6 × 10^5^ copies/mL) for 24 h. At 72 h post-pseudoviral inoculation, relative light units (RLUs) were detected using luciferase reporter assay and cell viability was measured using CCK-8 assay. These data were normalized to that of control DMSO. (G) Calu-3-hACE2 and (H) A549-hACE2 cells were pre-treated with 10 μM MDL28170, 10 μM CA074-Me, 10 μM E64d, or control DMSO for 2 h, followed by infection with SARS-CoV-2-S-pseudotyped viruses for 24 h. At 72 h post-pseudoviral inoculation, the fluorescence intensity of green fluorescence protein (GFP) was analysed by confocal microscopy. Scale bar: 10 μm; magnification: 630×. Calu-3-hACE2 (I) and A549-hACE2 (J) cells treated with 0.2 μM Oba and 2 μM or 10 μM MDL28170 for 72 h and infected with SARS-CoV-2-S-pseudotyped viruses as (C–F). RLUs were measured at 72 h post-pseudovirus inoculation. All experiments were repeated at least three times. * *P* < 0.05; ** *P* < 0.01; ns, not significant.

Cathepsin, including cathepsin B (CTSB) and cathepsin L (CTSL), are a class of endosomal cysteine proteases that mediate the cleavage of S protein in the acidic endosomal/lysosomal compartments, which is an essential step for viral entry into host cells [27]. CTSL is the major protease that cleaves SARS-CoV-2 S protein after viral entry in the intracellular endosomes [28]. Thus, the effect of obatoclax on CTSB and CTSL expression and maturation in Calu-3-hACE2 and A549-hACE2 cells was evaluated by western blotting. The results showed that obatoclax significantly decreased the expression of mature CTSL (29 kDa), but had no significant effect on the expression of mature CTSB (38 kDa) (Figure 4(A,B); Fig. S5). Additionally, we found that obatoclax did not cause an obvious change of other enzyme dynamin expression in Calu-3-hACE2 and A549-hACE2 cells (Fig. S4). To further assess the relevance of cathepsins, Calu-3-hACE2 and A549-hACE2 cells were infected with the S-pseudovirus in the presence and absence of inhibitors specific for CTSB (CA074-Me) or CTSL (MDL28170). In agreement with the aforementioned findings, we found that the CTSL inhibitor had a significant inhibitory effect on S-mediated entry (Figure 4(C,D)), while the CTSB inhibitor did not (Figure 4(E,F)). Accordingly, immunofluorescence microscopy showed that the inhibition of CTSL significantly decreased the abundance of SARS-CoV-2 S compared to control cells, while CTSB inhibition again did not show significant effects (Figure 4(G,H)). Additionally, after Calu-3-hACE2 and A549-hACE2 cells were treated with obatoclax and 2 or 10 μM of the CTSL inhibitor, no additive effects were observed (Figure 4(I,J)), suggesting an inhibitory mechanism along overlapping pathways. Taken together, these data strongly suggested that obatoclax affects SARS-CoV-2 entry by impairing CTSL activity.

### Obatoclax blocks the membrane fusion of SARS-CoV-2 by inhibiting furin activity

**Figure 5.**
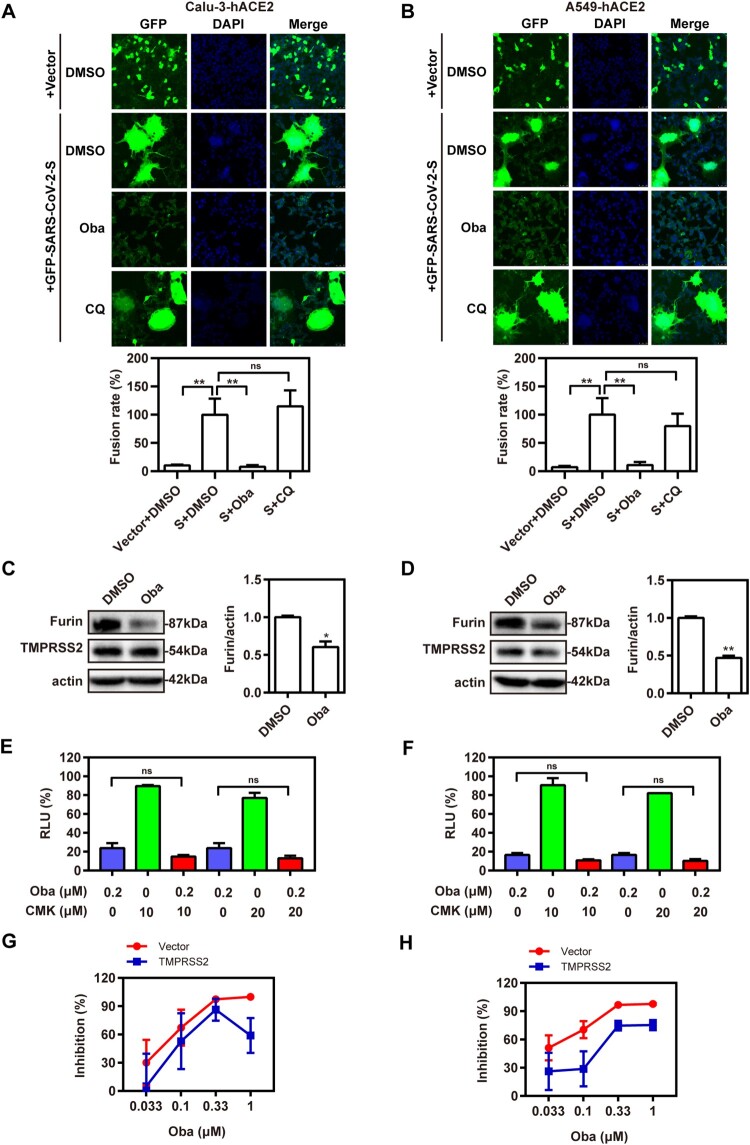
Obatoclax blocks the membrane fusion of SARS-CoV-2 by inhibiting protease furin activity. HEK293T cells as effector cells were transfected with plasmid pS-G614; Calu-3 (A) and A549 (B) cells were transfected with plasmid hACE2 as target cells. The cell–cell fusion rate was analysed by confocal microscopy in the presence of 0.2 μM Obatoclax (Oba) or DMSO. Scale bar: 10 μm; magnification: 200×. The fusion rate was set as 100% in a DMSO-treated group. Calu-3-hACE2 (C) and A549-hACE2 (D) cells were treated with 0.2 μM Oba or DMSO for 48 h. The levels of furin and TMPRSS2 from cell lysates were measured by western blotting, using beta-actin as a loading control. The furin /actin ratio was calculated by densitometry analysis. Calu-3-hACE2 (E) and A549-hACE2 (F) cells were treated with 0.2 μM Oba and 10 μM or 20 μM Decanoyl-RVKR-CMK (CMK). Calu-3 (G) and A549 (H) cells were co-transfected with plasmid hACE2 and TMPRSS2 or vector control pCMV3, and then treated with the indicated concentrations (0.033, 0.1, 0.33, and 1 μM) of Oba for 72 h. At 72 h post-SARS-CoV-2-S-pseudovirus inoculation (7.6 × 10^5^ copies/mL), relative light units (RLUs) were measured using luciferase reporter assay and normalized to that of control DMSO. All experiments were repeated at least three times. * *P* < 0.05; ** *P* < 0.01; ns, not significant.

Membrane fusion is obviously indispensable for SARS-CoV-2 to initiate its replication cycle [6]. In order to evaluate how effective obatoclax is against membrane fusion-mediated entry of SARS-CoV-2, cell–cell fusion assay was performed. We observed that obatoclax diminished the S-induced cell–cell fusion rates of Calu-3-hACE2 and A549-hACE2 cells at ∼90%, respectively (Figure 5(A,B)). Consistent with the study of Hoffmann et al. [18], chloroquine did not inhibit syncytia formation, which may explain the limited antiviral effect of chloroquine in relevant human cells. Collectively, our data showed that obatoclax inhibits the membrane fusion-mediated entry induced by the SARS-CoV-2-encoded S protein.

During SARS-CoV-2 entry into target cells, the S protein initially binds to hACE2 followed by its cleavage by furin at the S1/S2 junction and a second cleavage event at the S2 site catalysed by TMPRSS2 [13,29–32]. To investigate whether obatoclax also affects the activity of critical proteases related to S protein-mediated membrane fusion, we determined TMPRSS2 and furin expression in Calu-3-hACE2 and A549-hACE2 cells treated with obatoclax. The furin expression was significantly decreased in obatoclax-treated cells, whereas no apparent effect on TMPRSS2 was observed (Figure 5(C,D)). Thus, the inhibitory effect on furin activity may contribute to the antiviral activity of obatoclax. To test this hypothesis, Calu-3-hACE2 and A549-hACE2 cells were treated with the furin inhibitor decanoyl-Arg-val-Lys-Arg-CMK in combination with obatoclax. We did not observe significant additive effects on S-mediated entry, arguing in favour of the notion that furin is involved in the obatoclax-sensitive mechanism affecting the membrane fusion (Figure 5(E,F)). Additionally, to evaluate the inhibitory effect of obatoclax on SARS-CoV-2 infection in the lung cells with TMPRSS2 overexpression, we transfected the TMPRSS2 plasmid or an empty vector into Calu-3-hACE2 and A549-hACE2 cells. The luciferase reporter assay revealed that obatoclax still decreased SARS-CoV-2 infection by approximately 30% compared with control group (Figure 5(G,H)), indicating that SARS-CoV-2 infection may depend on several proteases, including furin and TMPRSS2. Collectively, obatoclax inhibits membrane fusion-mediated SARS-CoV-2 entry by inhibiting furin activity.

### Obatoclax inhibits membrane fusion of SARS-CoV-2 by targeting MCL-1

**Figure 6.**
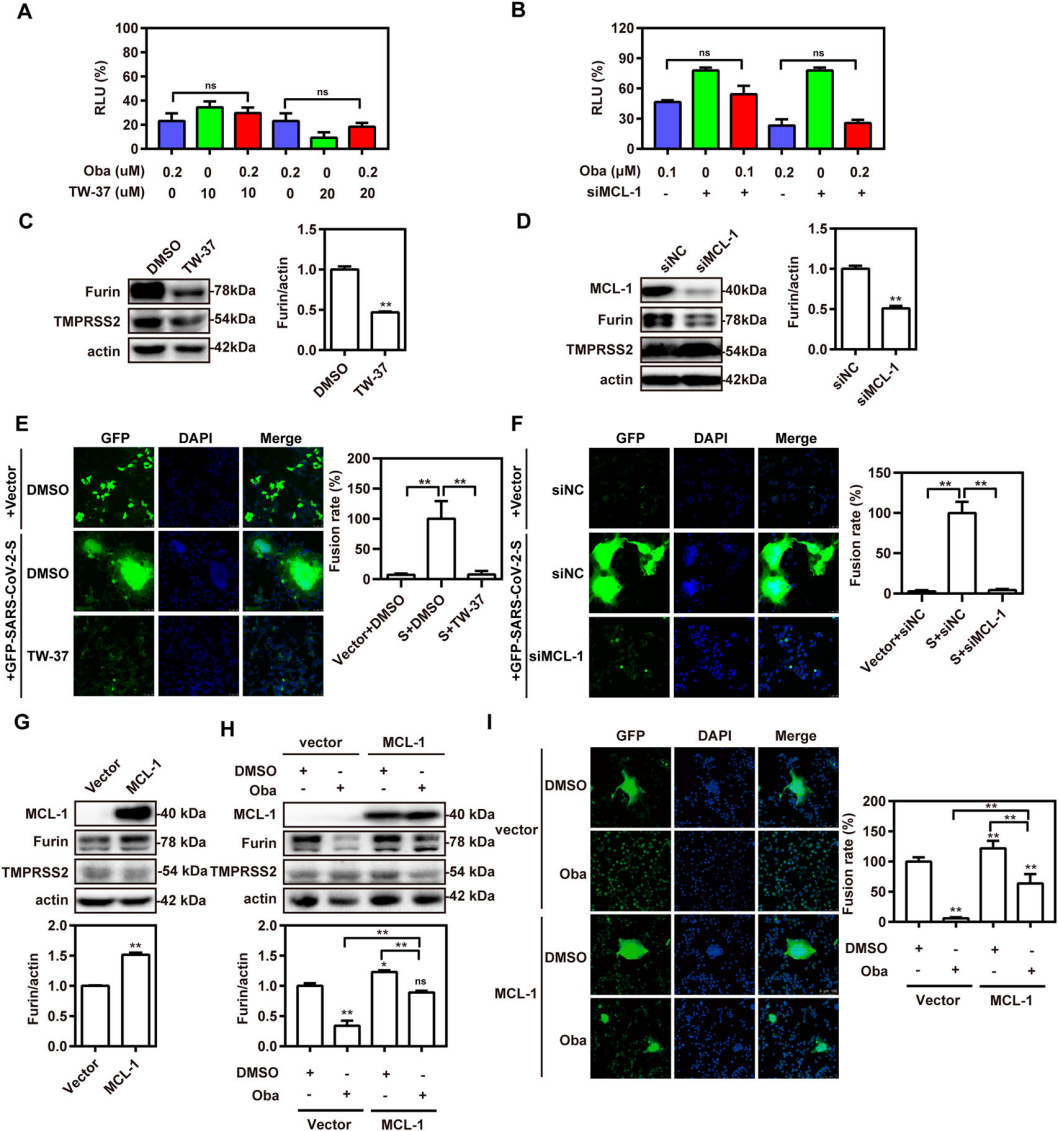
Obatoclax inhibits membrane fusion of SARS-CoV-2 by targeting MCL-1. (A) A549 cells were pre-transfected with plasmid hACE2. At 72 h post transfection, A549-hACE2 cells were treated with 0.2 μM obatoclax (Oba) and 10 μM or 20 μM TW-37 for 2 h, followed being infected with SARS-CoV-2-S-pseudotyped viruses (7.6 × 10^5^ copies/mL). (B) A549 cells were pre-transfected with plasmid hACE2 and specific siRNA against MCL-1 (siMCL-1) or siRNA negative control (siNC), then treated with 0.1 μM or 0.2 μM Oba for 72 h. At 72 h post-pseudovirus inoculation, relative light units (RLUs) were measured using luciferase reporter assay and normalized to that of control DMSO. (C) A549-hACE2 cells were treated with 10 μM TW-37 or control DMSO for 48 h and total protein was extracted for western blotting. (D) A549-hACE2 cells were transfected with 40 nM siMCL-1 or siNC for 48 h. The levels of furin and TMPRSS2 from cell lysates were measured by western blotting, and β-actin was used as a loading control. HEK293T cells as effector cells were transfected with plasmid pS-G614, and A549-hACE2 cells were used as target cells. (E) The effect of TW-37 on cell–cell fusion was imaged by confocal microscopy. (F) The effect of MCL-1 silencing on cell-cell fusion was imaged by confocal microscopy. Scale bar: 10 μm; magnification: 200×. (G) A549 cells were transfected with a plasmid MCL-1 expressing tag protein Flag or vector control pCMV-10. At 48 h post transfection, total protein was extracted for western blot. A549 cells were pre-transfected with plasmids hACE2 and a clone vector expressing MCL-1 with tag protein Flag or vector control pCMV-10. At 72 h post transfection, followed by treatment with 0.2 μM Oba or DMSO for 48 h. (H) The levels of furin and TMPRSS2 from cell lysates were measured by western blotting. (I) The effect of MCL-1 overexpression and Oba treatment on the cell-cell fusion was imaged by confocal microscopy. All the experiments were repeated independently at least three times. * *P* < 0.05; ** *P* < 0.01; ns, not significant.

Obatoclax, a novel BCL-2 homology domain-3 mimetic, is a small molecule antagonist of pan-BCL-2 family of proteins, including MCL-1 [33]. It has been demonstrated that the anti-influenza A virus (IAV) activity of obatoclax is associated with its capacity to inhibit MCL-1 [34]. Therefore, we addressed whether this ability is also effective against SARS-CoV-2 infections. To determine if MCL-1 is necessary for SARS-CoV-2 entry, we treated Calu-3-hACE2 and A549-hACE2 cells with a specific MCL-1 inhibitor (TW-37) in the presence or absence of obatoclax. The MCL-1 inhibitor showed potent antiviral activity. However, the combined treatment did not improve the antiviral activity compared to individual treatment regimens (Figure 6(A); Fig. S6A), pointing towards overlapping mechanisms. Next, we applied specific siRNA to ablate MCL-1 expression in the Calu-3-hACE2 and A549-hACE2 cells, which were subsequently treated with obatoclax. Although decreased MCL-1 levels diminished the S-mediated entry, we again did not observe significant additive effects in the S-pseudovirus system (Figure 6(B); Fig. S6B), suggesting that MCL-1 is a target of obatoclax in terms of the inhibition of SARS-CoV-2 infection. Furthermore, we investigated the relationship between MCL-1 protein and the membrane fusion pathway of SARS-CoV-2 entry. Western blotting showed that the level of furin expression in Calu-3-hACE2 and A549-hACE2 cells was significantly reduced after MCL-1 inhibitor treatment, whereas no difference was observed in TMPRSS2 expression (Figure 6(C); Figs. S5 and S6C). Accordingly, MCL-1-ablating siRNAs significantly decreased furin expression in both cell lines (Figure 6(D); Fig. S6D). Finally, we performed the aforementioned cell–cell fusion assays applying the MCL-1 inhibitor (Figure 6(E); Fig. S6E) or MCL-1 ablation by siRNAs (Figure 6(F); Fig. S6F). In both cases and in Calu-3-hACE2 and A549-hACE2 cells, the fusogenic activity of the SARS-CoV-2 S protein was reduced by approximately 90%. In contrast, we found that MCL-1 overexpression increased the levels of furin expression but not TMPRSS2 expression in A549-hACE2 cells (Figure 6(G)). Although MCL-1 overexpression did not completely reverse the inhibitory effect of obatoclax on furin expression (Figure 6(H)) and SARS-CoV-2-S-mediated syncytium formation (Figure 6(I); Fig. S6G), it obviously weakened its effects. Collectively, our results indicated that obatoclax inhibits the S protein-mediated membrane fusion pathway of SARS-CoV-2 entry by targeting MCL-1.

### Obatoclax inhibits SARS-CoV-2-encoded S proteins including those derived from variants of concern

**Figure 7.**
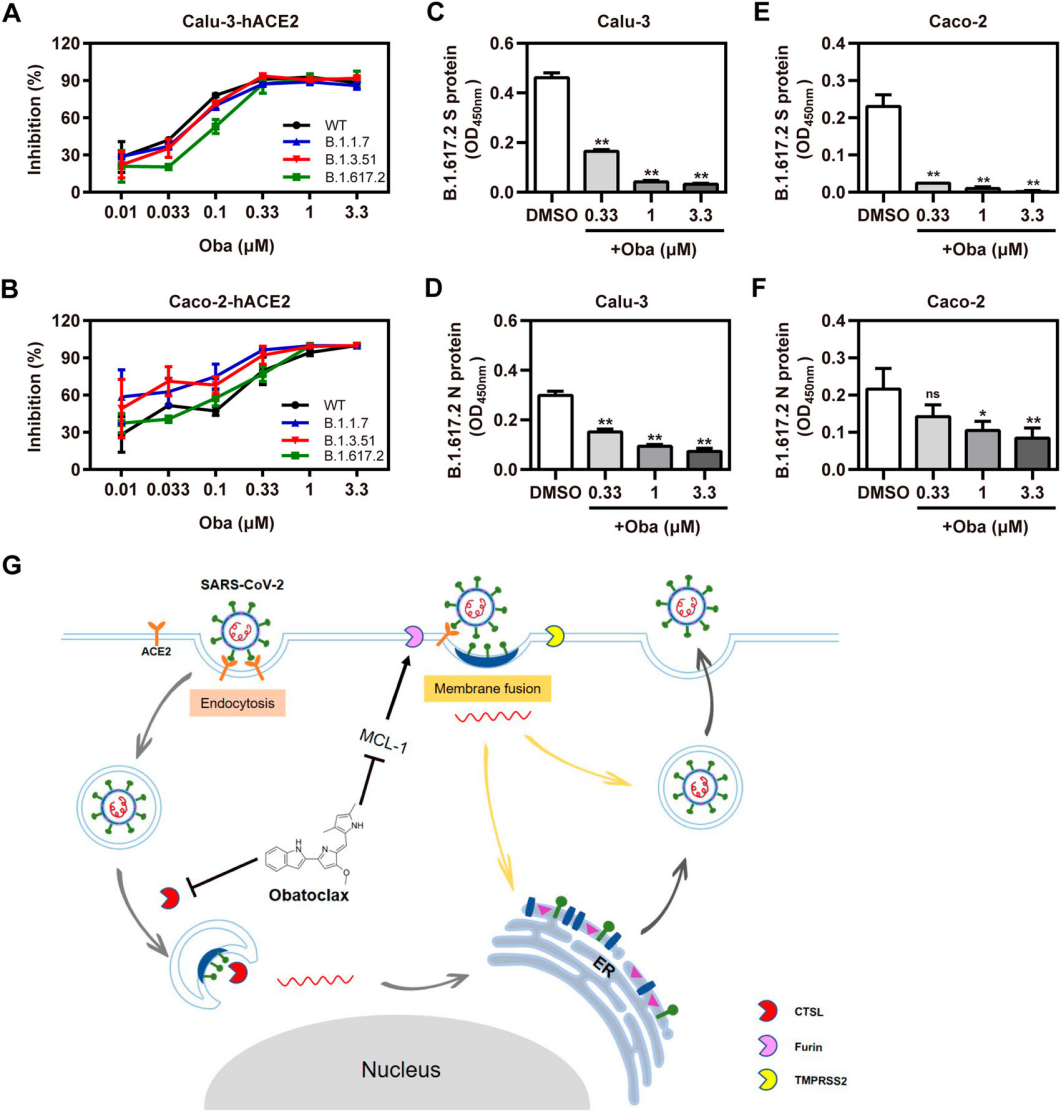
Obatoclax inhibits different mutant SARS-CoV-2 infections *in vitro.* Calu-3 and Caco-2 cells were pre-transfected with plasmid hACE2. At 72 h post transfection, Calu-3-hACE2 (A) and Caco-2-hACE2 (C) cells were treated with different concentrations (from 0.01 to 3.3 μM) of obatoclax (Oba) for 2 h, followed being infected with equivalent doses (7.6 × 10^5^ copies/mL) of each SARS-CoV-2-S-pseudotyped viruses, including wild-type (WT; B.1, GenBank: QHD43416), Alpha (B.1.1.7), Beta (B.1.351), and Delta (B.1.617.2) mutant spike pesudotyped viruses. Relative light units (RLUs) were measured using luciferase reporter assay and normalized to that of control DMSO. These experiments were repeated at least three times. (C–F) Calu-3 and Caco-2 cells were pre-treated with Oba at 0.33, 1 μM, and 3.3 μM for 1.5 h and then infected with a clinical SARS-CoV-2 isolate variant Delta (B.1.617.2) (MOI  = 0.1). At 2 h post-infection, the supernatant was removed and replaced by fresh culture medium containing the respective amount of obatoclax. At 24 h post-virus inoculation, viral S (C,D) and N (E,F) proteins in the infected cells were quantified by in-cell-ELISA. These experiments were repeated at least once. (G) Proposed model of Oba inhibition of SARS-CoV-2 infection by blocking endocytosis through inhibiting endosomal cysteine protease CTSL activity and SARS-CoV-2-S-mediated membrane fusion of viral entry through inhibiting furin protease activity by targeting MCL-1.

Recently, Variants of Concern (VOC), such as Alpha (B.1.1.7) (also called 501Y.V1; initially identified in the UK), Beta (B.1.351) (also known as 501Y.V2; initially identified in South Africa), and Delta (B.1.617.2) (also known as 478 K.V1; initially identified in India), emerged and rapidly became dominating variants in certain regions [35–37]. Therefore, we evaluated whether obatoclax also executes inhibitory effects against S proteins derived from SARS-CoV-2 VOC. In accordance with the aforementioned findings, obatoclax exhibited a pan-variant inhibitory activity against S-mediated entry, in human Calu-3-hACE2, Caco-2-hACE2, and A549-hACE2 cells (Figure 7(A,B); Fig. S7). To further evaluate the real antiviral activity of obatoclax against the infections with the clinical SARS-CoV-2 isolate variant Delta, the icELISA was applied to measure the levels of the viral S and N protein in infected Calu-3-hACE2 and Caco-2-hACE2 cells treated with indicated graded obatoclax concentrations (0.33–3.3 μM). Obatoclax significantly and dose-dependently diminished the viral S (Figure 7(C,D)) and N antigen (Figure 7(E,F)) in SARS-CoV-2 Delta-infected cells. Therefore, obatoclax should be further explored in clinical studies as broad-spectrum and pan-variant inhibitor of SARS-CoV-2 infections.

## Discussion

The ongoing COVID-19 pandemic rapidly spread around the globe, creating profound crises in health, economic, and social sectors. There is an urgent unmet need for effective, safe, and affordable SARS-CoV-2 therapies [38]. Herein, we presented ample evidence that obatoclax, which has been applied in patients in oncologic clinical trials where it was termed safe [39,40], elicits effective anti-SARS-CoV-2 activity at nanomolar, non-toxic concentrations in various human cells and against authentic clinical SARS-CoV-2 isolates.

Compared to the development of new antiviral therapeutic compounds, drug repurposing for COVID-19 represents a valuable strategy due to its lower costs and faster approval time. Obatoclax, a synthetic indole bipyrrole derivative of bacterial prodigiosin, was originally developed as an anti-cancer agent that inhibits the Bcl-2 family of pro-survival proteins [17]. Obatoclax underwent phase I and II clinical trials as a treatment option for various leukaemia entities and solid tumours, such as acute myeloid leukaemia, chronic lymphocytic leukaemia, and small cell lung cancer [41]. It has also been shown that obatoclax displayed BCL-2 associated x protein/BCL-2 antagonist killer (BAX/BAK) dependent anti-cancer efficacy by inducing mitochondrial apoptosis [42]. Additionally, some parasites, such as schistosomes, *Trypanosoma brucei*, and apicomplexan parasites, exhibited a sensitive and strong response to obatoclax, highlighting the possible functional roles of obatoclax in clinical application [43]. Obatoclax also inhibits the replication of different viruses, such as IAV, herpes simplex virus 2, echovirus, and Zika virus, which is independent of its pro-apoptotic activity [44,45]. Recently, obatoclax was reported to inhibit the SARS-CoV-2 infection in non-human primate (NHP)-derived Vero E6 and human nasal epithelial cells [46,47]. However, to our knowledge, the molecular mechanism behind the antiviral activity was unknown prior to this work. The knowledge concerning the exact molecular mechanisms is usually a prerequisite for clinical approval considering that it supports the assessment of effective trough levels, potential side effects, and resistance mechanisms.

SARS-CoV-2 enters host cells either through fusion with plasma surface membranes (“early pathway”) or endo-/lysosomal membranes (“late pathway”). Obatoclax elicits a multi-pronged attack on SARS-CoV-2 (Figure 7(G)). Firstly, it acts by inhibiting endocytosis during SARS-CoV-2 entry by neutralizing endo-/lysosomal acidification. Certain coronaviruses, such as SARS-CoV, MERS-CoV, and IBV, utilize the endocytic/lysosomal pathway for their entry into host cells [9,10,48]. During the infection process, endocytosis mediated by endosomal cathepsins is a critical step for viral entry [21]. Similar findings were reported in other papers in the context of studies addressing other viruses such as alphaviruses, which also utilize acidic late endosomes/multi-vesicular bodies for entry by changing the pH in acidic endosomes [17,44]. In our study, we observed that obatoclax treatment caused an obviously increased LAMP1 expression and a significant inhibition of viral entry by increasing the lysosomal pH, as evidenced by the pH-sensitive Lysotracker Red stain.

Previous studies have revealed that lysosomal stress or suppression of lysosome function may induce a feedback of inhibiting MTOR complex 1 (MTORC1) activity [7,49], which may activate the transcription factor EB (TFEB, a master regulator of lysosomal biogenesis) to enhance related lysosomal gene expression (including LAMP1) [50,51]. Thus, we assumed that the increased LAMP1 expression in the cells with obatoclax treatment would be caused by the elevation of lysosomal pH by inhibiting MTORC1 activity and activating TFEB activity. Cathepsins are highly abundant in lysosomes and play a critical role during SARS-CoV-2 entry. In particular, CTSL, which has an optimum activity in acidic environments, has been shown to represent the major protease cleaving the S1 subunit of the extinct, first SARS-CoV, triggering the fusion of viral and host membranes. CTSL has been implicated in the entry of SARS-CoV-2, which is closely related to SARS-CoV [28,52–54]. Our results showed that obatoclax significantly reduces the activity of CTSL by altering endosomal acidification. Therefore, the blockage of the endocytosis of SARS-CoV-2 entry in the cells with obatoclax treatment would be caused by elevating the lysosomal pH by inhibiting cathepsin (mainly CTSL) activity.

For SARS-CoV-2 entry into hosts cells, proteases such as furin and TMPRSS2 are required [55,56]. TMPRSS2 cleaves SARS-CoV-2 S at the S2 site and furin cleaves the viral S protein at the S1/S2 junction. Both proteases are required for viral entry into human lung cells [57]. In contrast to most other beta-coronaviruses of subtype B, only the SARS-CoV-2 S protein contains a furin cleavage site, which is considered to contribute to its enhanced infectivity and increased membrane fusion efficiency in humans, compared to other closely related coronaviruses with lower pandemic potential [13,58]. Accordingly, Cheng et al. and Wu et al. reported that certain furin inhibitors could be applied as potential antiviral agents to control SARS-CoV-2 infection and pathogenesis [59,60]. As a second relevant mechanism of antiviral activity against SARS-CoV-2, we found that obatoclax treatment decreased the abundance of the furin protein. Thirdly, as a novel small molecule antagonist of MCL-1 [33], our results further showed that obatoclax inhibits the membrane fusion of SARS-CoV-2 by targeting MCL-1 in the context of SARS-CoV-2 infection. Our findings are consistent with the inhibitory effect of obatoclax against IAV infection [34].

SARS-CoV-2 VOC can become resistant against post-exposure prophylactic approaches such as monoclonal antibodies [61]. Using the S-pseudovirus system, we showed that obatoclax retained its potency against highly relevant S variants such as the S proteins of VOCs Alpha, Beta, and Delta [35–37], suggesting broad pan-variant activity against different SARS-CoV-2 isolates. These findings were verified by infection experiments using a clinical SARS-CoV-2 Delta virus isolate.

Taken together, obatoclax shows a strong antiviral effect by inhibiting SARS-CoV-2 entry *in vitro.* Its inhibitory effect depends on the blockage of both endocytosis and membrane fusion pathways essential for viral entry. Therefore, obatoclax is a novel, valuable repurposable drug candidate for the treatment of SARS-CoV-2 infections and deserves to be explored in clinical trials.

## Supplementary Material

Supplemental Material
