# Supplementary material for: Obatoclax inhibits SARS-CoV-2 entry by altered endosomal acidification and impaired cathepsin and furin activity in vitro
Source: Emerg Microbes Infect. 2022 Feb 10;11(1):483–97. doi: 10.1080/22221751.2022.2026739 (PMC8843317; doi:10.1080/22221751.2022.2026739)
Supplement: Supplemental Material [file TEMI_A_2026739_SM1540.docx]

**Supplemental materials and methods**

***Cell transfection***

A total of 1.5×10^5^ per well Calu-3, A549, or Caco-2 cells were seeded into 12-well plates 1 day before transfection. When cells reached 80–90% confluence, they were transfected with plasmids or siRNAs using Lipofectamine 3000 Transfection Reagent (Invitrogen, Carlsbad, CA, USA; L3000015) according to the manufacturer’s instructions. After 4-6 h, cells were transferred to fresh complete medium and incubated at 37°C in a humidified atmosphere contained 5% CO_2_. The siRNAs used in this study were purchased from GenePharma (Shanghai GenePharma, China). The siRNAs sequences were as follows: siRNA negative control (siNC), sense: UUCUCCGAACGUGUCACGUTT; antisense: ACGUGACACGUUCGGAGAATT. siMCL-1 #1, sense: GUGCCUUUGUGGCUAAACATT; antisense: UGUUUAGCCACA AAGGCACTT.

***In-cell ELISA (icELISA)***

SARS-CoV-2 S and N proteins were detected by icELISA, as previously described [1]. Briefly, Calu-3 and Caco-2 cells were seeded into 96-well microplates 2 days before infection. The drugs were added directly into the medium, at the concentrations previously mentioned, and cells were incubated for 1.5 h before SARS-CoV-2 infection was performed. At 2 h post-infection, the supernatant was removed and replaced by fresh medium containing the respective amount of drug. At 24 h post-infection, cells were fixed with 4% (w/v) paraformaldehyde/PBS. Cells were permeabilized with 1% (v/v) Triton-X-100/PBS and blocked with 3% (v/v) FCS/PBS. The primary antibodies were diluted 1:10,000 for icELISA detection and incubated for 2 h at 25 °C or overnight at 4°C. Peroxidase-labelled secondary antibody was incubated for 1 to 2 h. Washing steps were performed with 0.05% (v/v) Tween-20/PBS. Tetramethylbenzidin (TMB) substrate was added to visualize the enzyme reaction. The reaction was stopped with 0.5 M HCl. Finally, absorbance was recorded at 450 nm using a microplate multireader (Mithras2 LB 943; Berthold Technologies). An α-S mAb (kindly provided by Peter Miethe, fzmb, Bad Langensalza, Germany), α-N mAb (ABIN6952435), and POD-coupled secondary antibodies (Dianova) were used.

***Quantitative reverse-transcription (RT-q) PCR***

SARS-CoV-2 progeny was analyzed by quantification of viral RNA extracted from the culture supernatants of infected cells using the INSTANT Virus RNA/DNA Kit (Analytik Jena, Jena, Germany). RNA was quantified by diagnostic RT-qPCR targeting the SARS-CoV-2 genes S and E (RealStar® SARS-CoV-2 RT-PCR kit, Altona, Hamburg, Germany). The primers used for quantification of cytokine and chemokine expression were IL-6 forward 5′-ACTCACCTCTTCAGAACGAATTG-3′, reverse 5′-CCATCTTTGGAAGGTTCAGGTTG-3′; IL-10 forward 5′-GACTTTAAGGGTTACCTGGGTTG-3′, reverse 5′-TCACATGCGCCTTGATGTCTG-3′; IP-10 forward 5′-GTGGCATTCAAGGAGTACCTC-3′, reverse 5′-TGATGGCCTTCGATTCTGGATT-3′; MCP-1 forward 5′-CAGCCAGATGCAATCAATGCC-3′, reverse 5′-TGGAATCCTGA ACCCACTTCT-3′; MIP1α forward 5′-AGTTCTCTGCATCACTTGCTG-3′, reverse 5′-CGGCTTCGC TTGGTTAGGAA-3′; β-actin forward 5′-ATCGTGCGTGACATTAAGGAG-3′, reverse 5′-GGAA GGAAGGCTGGAAGAGT-3′.

***Confocal microscopy***

Immunofluorescence staining was carried out as previously described [2]. Briefly, cells were fixed in 4% (w/v) paraformaldehyde for 10 min at 25 °C and permeabilized with 0.1% (v/v) Triton X-100 (Sigma, T8787) for 10 min at 25 °C. For nuclear staining, cells were treated with 1 mg/mL 4’,6-diamidino-2-phenylindole (DAPI, Sigma, D9542) for 10 min. Additionally, the LysoTracker Red (Thermo Fisher, L12492) staining was performed according to the protocol that we previously described [3]. Stained sections were analyzed using a laser scanning confocal microscope (Leica TCS SP8, Solms, Germany).

***Immunoblot analysis***

Western blot analysis was performed as previously described [2]. Primary antibodies dilutions were used as follows: ACE2 (Proteintech, 21115-1-AP, 1:1,000), CD63 (sc-5275, Santa Cruz, 1:1,000), cathepsin B (CTSB; Proteintech, 12216-1-AP, 1:1,000), cathepsin L (CTSL; Proteintech, 10938-1-AP, 1:1000), dynamin 1 (Proteintech, 18205-1-AP, 1:1,000), EEA1 (Proteintech, 66218-1-Ig, 1:1,000), furin (Proteintech, 18413-1-AP, 1:1,000), LAMP1 (9091, Sigma, 1:1,000), MCL-1 (Proteintech, 16225-1-AP, 1:1,000), TMPRSS2 (Proteintech, 14437-1-AP, 1:1,000), and β-actin (Proteintech, 66009-1-Ig, 1:5,000). The antibody against SARS-CoV-2 S was kindly provided by Prof. Xuefei Cai (Chongqing Medical University). Membranes were incubated with peroxidase-conjugated rabbit anti-mouse IgG antibody (Jackson ImmunoResearch, 315-035-048, 1:15,000) or Peroxidase-AffiniPure goat anti-rabbit IgG antibody (Jackson ImmunoResearch, 111-035-045, 1:15,000). Protein bands were visualized using Clarity^TM^ Western ECL Substrate (Bio-Rad, 1705061) and quantified by densitometry using Image J software.

***Cytotoxicity assay***

Cell viability was assessed using the Cell Counting Kit-8 (CCK-8; MedChemExpress, HY-K0301) according to the manufacturer’s instructions. Briefly, a total of approximately 1.5×10^4^ cells were seeded in 96-well plates and incubated with different indicated concentrations of drugs for 72 h. Cell viability was determined by measuring the absorbance of each well at 450 nm in a microplate reader.

***Cell-cell fusion assay***

For fusion assays, approximately 3×10^6^ per well HEK293T cells (as effector cells,) were seeded into 6-well plates and transfected with plasmid pAdTrack-TO4-GFP encoding SARS-CoV-2 S protein using Lipofectamine 3000 (Invitrogen, Carlsbad, CA, USA). A total of 3×10^6^ per well Calu-3 or A549 cells (as target cells) were seeded into 6-well plates and overexpressed hACE2. At 24 h post-transfection, effector and target cells were co-cultured at 1:1 ratio in 6-well cell plates, covering 90% of the well surface. At 6 h after co-culture, cells were treated with 0.1 μM obatoclax or 2 μM chloroquine. Cell-cell fusion was measured using a laser scanning confocal microscope (Leica TCS SP8, Solms, Germany). For quantification of syncytia, each image was counted using fused and unfused cells; the fused cells were at least twice as large as the unfused cells. The percentage of syncytia was calculated as: (number of fused cells / number of total cells) × 100%.

**Supplemental references**

1. Scholer L, Le-Trilling VTK, Eilbrecht M, et al. A Novel In-Cell ELISA Assay Allows Rapid and Automated Quantification of SARS-CoV-2 to Analyze Neutralizing Antibodies and Antiviral Compounds. Front Immunol. 2020;11:573526.

2. Lin Y, Wu C, Wang X, et al. Glucosamine promotes hepatitis B virus replication through its dual effects in suppressing autophagic degradation and inhibiting MTORC1 signaling. Autophagy. 2020 Mar;16(3):548-561.

3. Wang X, Wei Z, Cheng B, et al. ER stress promotes HBV production by enhancing utilization of the autophagosome- multivesicular body axis. Hepatology. 2021 Sep 28.

**Supplemental Figure**


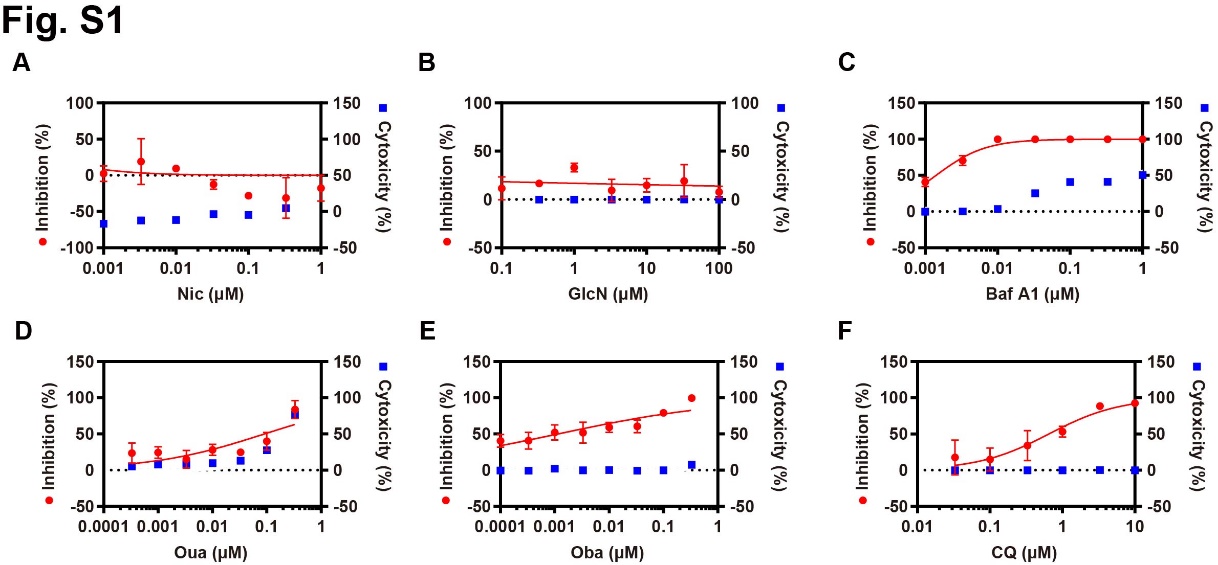


**Figure S1. Antiviral effect of six compounds in SARS-CoV-2 pseudovirus infected 293T-hACE2 cells.** HEK293T-hACE2 cells with stable ACE2 expression were pre-treated with different concentrations of niclosamide (Nic, A), glucosamine (GlcN, B), ouabain (Oua, C), bafilomycin A1 (Baf A1, D), obatoclax (Oba, E) and chloroquine (CQ, F) for 2 h, followed by infection with lentiviruses pseudotyped with SARS-CoV-2 spike protein. Relative light units (RLUs) were measured at 72 h post- pseudovirus inoculation. Cell viability was measured by CCK-8 assay. All these experiments were repeated at least three times.


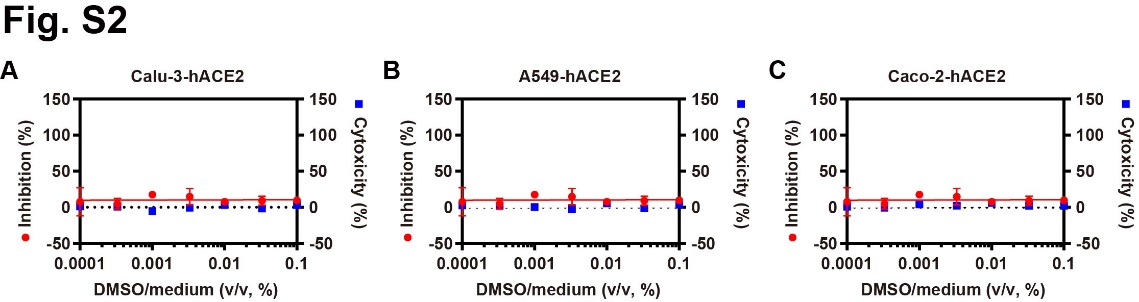


**Figure S2.** **cytotoxicity and anti-viral effect of DMSO in SARS-CoV-2-S pseudovirus infections.** A total of 1.5×10^4^ per well Calu-3-hACE2 (A), A549-hACE2 (B), or Caco-2-hACE2 (C) cells were seeded into 96-well plates and then treated with different volume ratios (from 0.0001% to 0.1%, v/v; corresponding to different concentrations (from 0.0033 μM to 3.3 μM) of obatoclax) of DMSO. At 72 h after pseudovirus inoculation, relative light units (RLUs) were detected using luciferase reporter assay and normalized to that of control mock treatment. Additionally, cell viability was measured using CCK-8 assay. All these experiments were repeated at least three times.


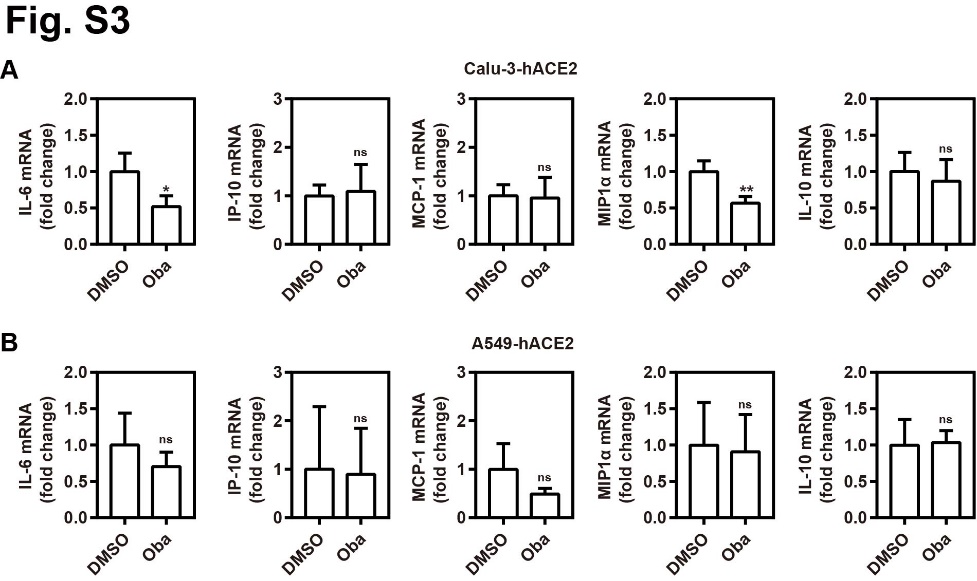


**Figure S3. Effect of obatoclax on pro-inflammatory cytokines associated to cytokine release syndrome.** Calu-3-hACE2 (A) and A549-hACE2 (B) cells were treated with 0.2 μM obatoclax (Oba). At 48 h after Oba treatment, total RNA was extracted by using TRIzol reagent and the mRNA levels of five pro-inflammatory cytokine expression was detected by real-time RT-PCR. β-actin was used as the internal control. * *P* < 0.05; ** *P* < 0.01; ns, not significant.


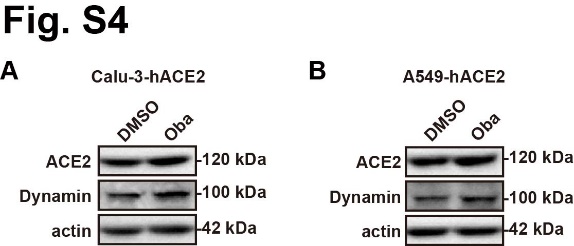


**Figure S4. Effect of obatoclax on ACE2 and dynamin expression.** Calu-3-hACE2 (A) and A549-hACE2 (B) cells were treated with 0.2 μM obatoclax (Oba). At 48 h after Oba treatment, the levels of ACE2 and dynamin were measured by western blotting.


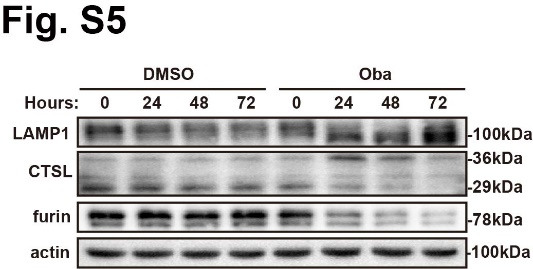


**Figure S5. Effect of obatoclax on LAMP1, CTSL and furin expression at different time points.** A549-hACE2 cells were treated with 0.2 μM obatoclax (Oba) or control DMSO for 24 h, 48h, and 72 h, respectively. The levels of LAMP1, CTSL and furin were measured by western blotting.


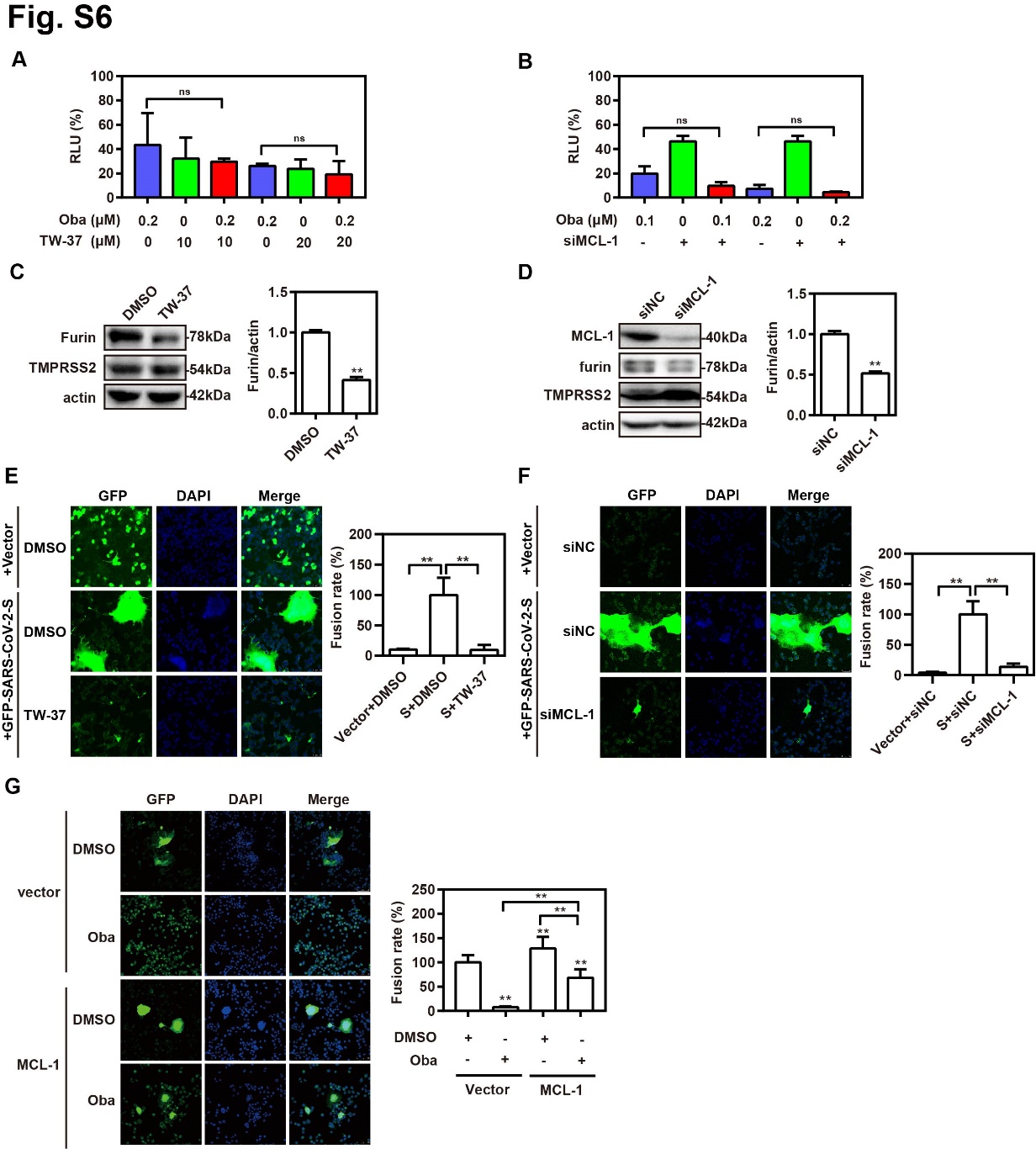


**Figure S6. Obatoclax inhibits membrane fusion of SARS-CoV-2 entry by targeting MCL-1 in Calu-3-hACE2 cells.** (A) Calu-3-hACE2 cells were treated with 0.2 μM obatoclax (Oba) and 10 μM or 20 μM TW-37 for 2 h and then infected with pseudotyped viruses. (B) Calu-3-hACE2 cells were pre-transfected with siRNA against MCL-1 and plasmid hACE2, then treated with 0.1 μM or 0.2 μM obatoclax for 72 h. At 72 h post-pseudovirus inoculation, relative light units (RLUs) were measured using luciferase reporter assay and normalized to that of control DMSO. (C) Calu-3-hACE2 cells were treated with 10 μM TW-37 or control DMSO for 48 h and total protein was extracted for western blotting. (D) A549-hACE2 cells transfected with 40 nM siMCL-1 or siRNA negative control (siNC) for 48 h. The levels of furin and TMPRSS2 were measured by western blotting. HEK293T cells as effector cells were transfected with plasmid pS-G614; Calu-3-hACE2 cells were used as target cells. (E) The effect of TW-37 on cell-cell fusion was imaged by confocal microscopy. (F) The effect of MCL-1 silencing on cell-cell fusion was imaged by confocal microscopy. (G) Calu-3-hACE2 cells were transfected with plasmid MCL-1 or vector control pCMV-10, followed by treatment with 0.2 μM Oba or DMSO for 48 h. The effect of on MCL-1 overexpression and Oba treatment on the cell-cell fusion was imaged by confocal microscopy. Scale bar: 10 μm; magnification: 200×. All these experiments were repeated independently at least three times. * *P* < 0.05; ** *P* < 0.01; ns, not significant.


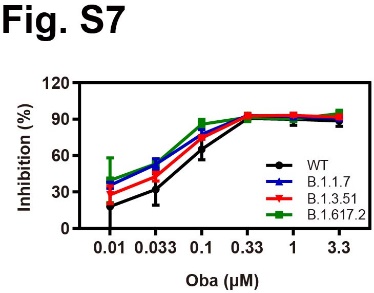


**Figure S7. Obatoclax inhibits different mutant S pseudotyped virus infections in A549-hACE2 cells*.*** A549-hACE2 cells were treated with the different concentrations of obatoclax (Oba) for 2 h, followed infection with equivalent doses of each pseudotype viruses, including wild-type (WT; B.1), Alpha (B.1.1.7), Beta (B.1.351), and Delta (B.1.617.2) mutant S pesudotyped viruses. At 72 h post pseudovirus inoculation, relative light units (RLUs) were measured using luciferase reporter assay and normalized to that of control DMSO. All these experiments were repeated at least three times.
